# In-situ micro-CT scanning and compressive strength assessment of diammonium hydrogen phosphate (DAP) treated chalk

**DOI:** 10.1038/s41598-023-43609-6

**Published:** 2023-10-05

**Authors:** Yevgeniy Samarkin, Abduljamiu Olalekan Amao, Murtada Saleh Aljawad, Mostafa Borji, Norman Scott, Murtadha J. AlTammar, Khalid M. Alruwaili

**Affiliations:** 1 Department of Petroleum Engineering, King Fahd University of Petroleum & Minerals, Dhahran, Saudi Arabia; 2 Center for Integrative Petroleum Research, King Fahd University of Petroleum & Minerals, Dhahran, Saudi Arabia; 3 Bruker microCT N.V, Kartuizersweg 3 B, 2550 Kontich, Belgium; 4 EXPECR Advanced Research Center, Saudi Aramco, Dhahran, Saudi Arabia

**Keywords:** Mineralogy, Natural gas

## Abstract

The occurrence of wellbore mechanical failure is a consequence of the interaction among factors such as in situ stress, rock strength, and engineering procedures. The process of hydrocarbons production, causing reduction of pore pressure, alters the effective stresses in the vicinity of a borehole, leading to borehole instability issues. Estimating the rocks’ elastic modulus and compressive strength is essential to comprehend the rock matrix’s mechanical response during drilling and production operations. This study aimed to assess the practicality of Diammonium Hydrogen Phosphate (DAP) application as a chemical for strengthening chalk in hydrocarbon reservoirs, to make it resistant to high stresses and failure during drilling and production. The mechanical and physical properties of Austin chalk rock samples treated with DAP under mimicked reservoir conditions were studied. The results showed that DAP is a highly effective carbonate rock consolidating agent that improves the mechanical strength of the chalk. Compressive test measurements conducted on rocks treated at two different temperatures (ambient and 50 °C) showed that DAP effectively strengthened the rock matrix, resulting in an increase in its compressive strength (22–24%) and elastic modulus (up to 115%) compared to the untreated sample. The favorable outcomes of this research suggest that the DAP solution holds promise as a consolidation agent in hydrocarbon reservoirs. This contributes to the advancement of knowledge regarding effective strategies for mitigating mechanical failures of the wellbore during drilling and production.

## Introduction

Ensuring wellbore stability is essential for enhancing safety, drilling efficiency, and reducing costs related to well construction and production operations. When drilling a well, the fundamental principle of wellbore stability highlights the importance of the wellbore walls withstanding the loading initially supported by the extracted rock^1^. The most common reasons for wellbore failure are the softness of the surrounding formation rock and its creeping behavior (plastic deformation), which results in the shrinkage of the wellbore^2–4^. As such, soft formations like chalk pose significant wellbore instability issues during drilling and production due to their complex geomechanics^2,5–8^. It has been reported that chalk formations experience viscous deformations under constant stresses, leading to the breakdown of the rock structure^2,9,10^. Common methods of wellbore instability prevention include extensive geomechanics modeling of the target formation and selection of appropriate drilling and completion strategies^11,12^. Moreover, recent studies in mitigating wellbore instability issues offer consolidation techniques through the controlled dissolution of glasses^13^. This work further expands on the idea of chemically consolidating formation rock to mitigate wellbore instability issues by treating chalk samples with consolidating agents commonly used in the preservation of cultural heritage^14–16^.

Carbonate rocks are frequently employed in cultural heritage structures and monuments for their durability and ornamental value^17–19^. Nevertheless, these rocks are prone to weathering and degradation over time, which may result in structural instability and aesthetic degradation^20^. Consolidation is a technique frequently applied to cultural heritage structures and utilized to improve carbonate rocks' durability and stability. The ultimate target of the consolidation is to heighten the rock’s strength and improve its ability to withstand the adverse impacts of the environment, such as weathering and erosion^21^. The common technique for consolidating carbonate rocks involves their treatment with chemical solutions^22^. Many chemicals, such as alkoxysilanes, phosphates, lime, and lime nanoparticles, have been tested in carbonate rock consolidation, and their strengthening capabilities were evaluated using different techniques^23–25^. For instance, tetraethyl orthosilicate (TEOS) was found to be especially effective in the consolidation of carbonate rocks containing some silicates in their mineralogy^21^. Aljawad et al.^26^ reported an increase of 18%, Graziani et al.^21^ achieved a 63% increase in dynamic modulus and a 126% increase in tensile strength, and Briffa and Vella^27^ observed a 20% improvement in drilling resistance of TEOS—treated limestone samples. Furthermore, several authors tested calcium hydroxide nanoparticles and calcium alkoxides on some carbonate samples and reported 5% to 30% improvement in ultrasound velocity measurements^28–31^. Desouky et al.^32^ and Samarkin et al.^33^ utilized zinc sulfate solution as consolidating agent and observed around 20% improvement in the hardness of limestone and chalk specimens assessed by the impulse hammering technique.

Many authors have confirmed experimentally that Diammonium Hydrogen Phosphate (DAP) is an extremely effective consolidating agent^14,15,34–37^. The consolidating principle of DAP is based on its reaction with CaCO_3_ and the generation of hydroxyapatite [Ca_10_(PO_4_)_6_(OH)_2_] minerals^38,39^. Possenti et al.^15^ demonstrated that the DAP reacts with calcium carbonate in a complex way and can form different intermediate phosphate phases that ultimately reprecipitate in the form of hydroxyapatite. Hydroxyapatite mineral is known to occur naturally and is an essential component of teeth enamel that helps prevent them from damage and decay^40,41^. Hydroxyapatite is a hard mineral, and its hardness is evaluated as five units according to the Mohs scale^42^. The consolidating process of DAP substance has been studied broadly. It was found that the degree of hardening by DAP solution is affected by the utilized solution’s concentration^43^. Sassoni et al.^43^ determined that the optimum concentration of the aqueous DAP solution required to obtain effective consolidation results is 1 M. Furthermore, significant improvements in the hardness of carbonate rocks treated with DAP solution have been reported. For example, Matteini et al.^44^ observed around 21% improvement in drilling resistance, Graziani et al.^21^ found a 22% increase in tensile strength, Murru and Fort^45^ observed a 460% enhancement in dynamic Young's modulus, and Sassoni et al.^16^ and Sena da Fonseca et al.^35^ recorded an increase in ultrasonic pulse velocity of up to 541% for various carbonate samples treated with DAP solution.

Recent studies tested consolidating efficiency of DAP at high-temperature conditions^14,46–48^. These studies demonstrated the hardening effect of DAP to be more significant if the rock samples are treated at high-temperature conditions. Samarkin et al.^48^ have claimed that more significant hardening could be a result of changes in the morphology and crystallinity of the hydroxyapatite minerals with the increase in the treatment temperature. An example of how the morphology, size, and hardness of hydroxyapatite minerals can be influenced by synthesis temperature is demonstrated in a number of studies. Manoj et al.^49^ showed that a temperature of 90 °C can cause HAP particles to form into nanorods during synthesis through a specific reaction. György et al.^50^ documented that HAP particle size is affected by synthesis temperature and raising the temperature from 80 °C to a higher temperature resulted in an increase in particle size. The hardness of HAP has been found to be related to its crystallinity and morphology by several authors^51–53^. Seyedmahmoud et al.^53^ found that deep dentin, a tooth tissue containing HAP, had smaller hardness due to an increase in carbonate content, which decreases HAP mineral crystallinity. Neira et al.^52^ used the nanoindentation technique to demonstrate that HAP's elastic modulus and hardness depend on the crystals' orientation. Finally, Hariani et al.^51^ demonstrated that Young's modulus and hardness of hydroxyapatite minerals raise with the increase in synthesis temperatures.

Measuring the compressive strength of rocks is crucial in petroleum engineering as it helps determine the reservoir's ability to withstand the pressures exerted during oil and gas production^54^. The compressive strength of rocks plays a significant role in designing wellbore stability, drilling programs, and hydraulic & acid fracturing operations in the petroleum industry^55^. Understanding the rocks’ compressive strength can prevent unexpected failures in producing wells, leading to safer and more efficient operations^56^. Previous studies on consolidating agents for hardening carbonate rocks were conducted using laboratory testing on samples that did not accurately reflect real downhole conditions^26,32,34,43,57^. This study aims to investigate how treating carbonate rocks with a Diammonium Hydrogen Phosphate solution affects their compressive strength and elastic modulus. The assessing rocks' compressive strength is done employing destructive techniques coupled with CT-scanning instead of the previously used non-destructive tests. The research expands the knowledge about the carbonate rocks’ mechanical behavior after their treatment with DAP solution, which can aid in introducing this technique in actual downhole operations to solve issues related to wellbore instability.

## Methodology

### Rock specimens’ preparation

**Figure 1:**
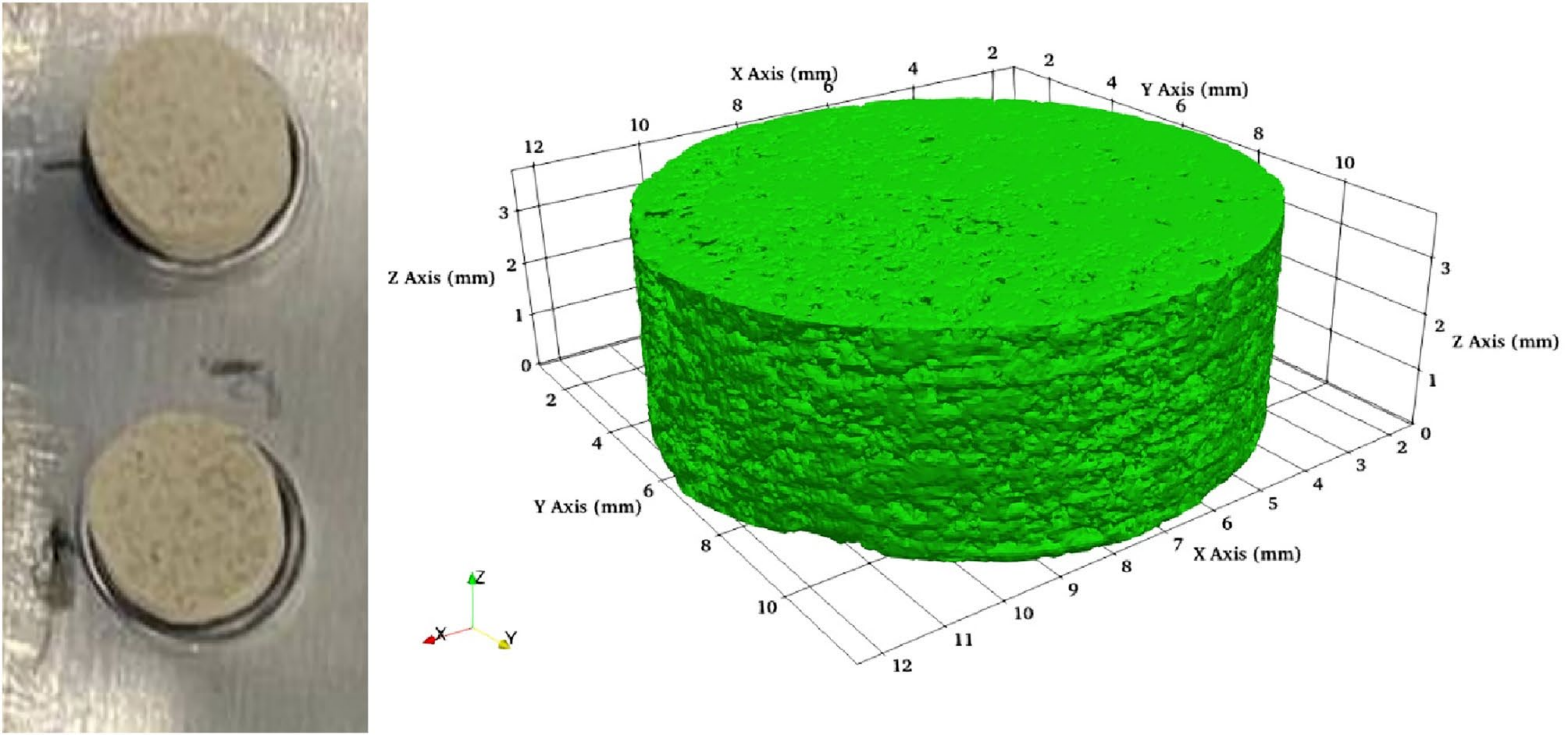
Cylindrical samples used in experiments and their 3D model with dimensions.

**Figure 2:**
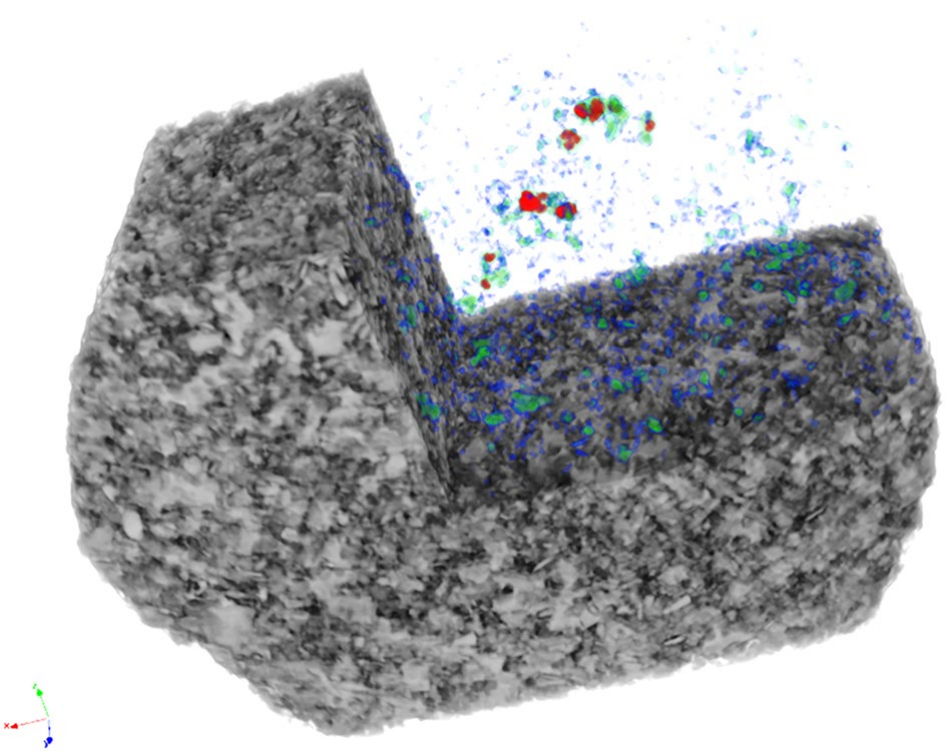
The pore space visualization of the tested samples.

**Figure 3:**
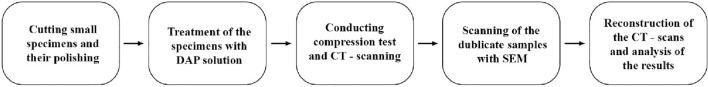
Experimental workflow**.**

A total of 3 Austin chalk samples with lengths of 3 inches and diameters of 1.5 inches were prepared for the experiments. One of these samples was left untreated, whereas others were treated with DAP at various conditions. The untreated and treated samples were then used to core small cylindrical specimens (Fig. 1). Several small cylindrical samples with dimensions and porosity (Fig. 2) values mentioned in Table 1 were utilized in testing and comparing their compressive strength. In contrast, others were studied by electron microscopy techniques to identify any changes in mineralogy that could happen due to the treatment with DAP solution. Figure 3 summarizes the entire experimental process followed in this study.

**Table 1:** Samples utilized in compressive strength testing.

Sample	Treatment	Radius 1 (mm)	Radius 2 (mm)	Area (mm^2^)	Height (mm)	Porosity (%)
CH1	Untr	10.83	10.83	368.47	3.75	21.14
CH2	Amb	10.82	10.48	356.24	6.6	24.91
CH3	50 °C	10.04	10.35	326.46	5.6	23.47

### Treatment conditions and procedures

The rock samples were treated at two different temperatures, namely 25 °C (further referred as ambient) and 50 °C, while maintaining ambient pressure. The specimens were treated by immersing them in glass beakers with 0.8 M DAP solution. For the high-temperature treatment, the glass beakers were placed in the oven and covered with specific glass caps that prevented the severe evaporation of the solution. Moreover, the beakers were topped up with deionized water from time to time to avoid changes in the concentration of the solution due to evaporation. The DAP solution was prepared by mixing 105.65 g of DAP salt with 1 L of deionized water. The solution was then equally distributed between the beakers to ensure no variation in its concentration. The samples aged in the DAP solution for 72 h, after which they were rinsed with deionized water to remove the salts.

### Analysis of morphology and mineralogy

The alteration in mineral composition was assessed by utilizing X-ray diffraction (Empyrean XRD, Malvern Panalytical), following the protocol and specifications outlined in Amao et al.^58^, and scanning electron microscopy SEM with a field emission gun (Zeiss Crossbeam 550) by contrasting pre- and post-treatment specimens. Briefly, the XRD technique involves irradiating a sample with X-rays at different angles. The X-rays leave the sample with specific intensity and at some diffraction angle, based on which the XRD pattern is built^59^. Each peak in the spectrum has its position (according to diffraction angle), which is matched with the existing database to identify the mineral. The SEM device utilizes the beam of electrons to scan the rock's surface, determine its morphology and generate the images^60^.

### Rock compressive strength measurements

The compression tests (uniaxial) were conducted at ambient temperature and pressure conditions on small cylindrical rock specimens using Material Testing Stage 3 (Bruker) mounted unto SKYSCAN 1275 micro-CT device (Bruker)^61^. This device allows for acquiring the CT—scan images of the samples while performing the compression test. The cylindrical rock specimens were subjected to a 1 × 10^−3^ mm s^−1^ deformation rate until the rock reached complete failure. Real-time data acquisition was utilized to obtain the correlation between the applied displacement and stress, resulting in the acquisition of the loading curve. The data acquired during compression was utilized in the calculation of compressive strength (UCS). The compressive strength of each specimen was found as the ratio of the maximum force at which that rock specimen failed by its cross-sectional area. Furthermore, Young's modulus of the samples was determined by calculating the gradient of the stress–strain curve obtained during the compression test experiments.

### Processing of CT-scan images

The datasets were reconstructed using Bruker NRecon software to provide a 3D digital representation of the samples, and the processing of the reconstructed CT images was performed using ImageJ software (v1.53). The image processing involved adjusting brightness, and contrast, and applying filters to remove artifacts. Furthermore, a thresholding algorithm was applied to segment the images and isolate the solid phase and pore space. The segmented images were used in basic characterization of the rock samples such as porosity calculation. The pore space extraction and visualization were done using Bruker 3D. SUITE Software. In addition, the processed images were used to generate 3D models of the samples, which were further processed in ParaView software for better visualization and representation.

## Results

### Changes in the mineralogy

**Figure 4:**
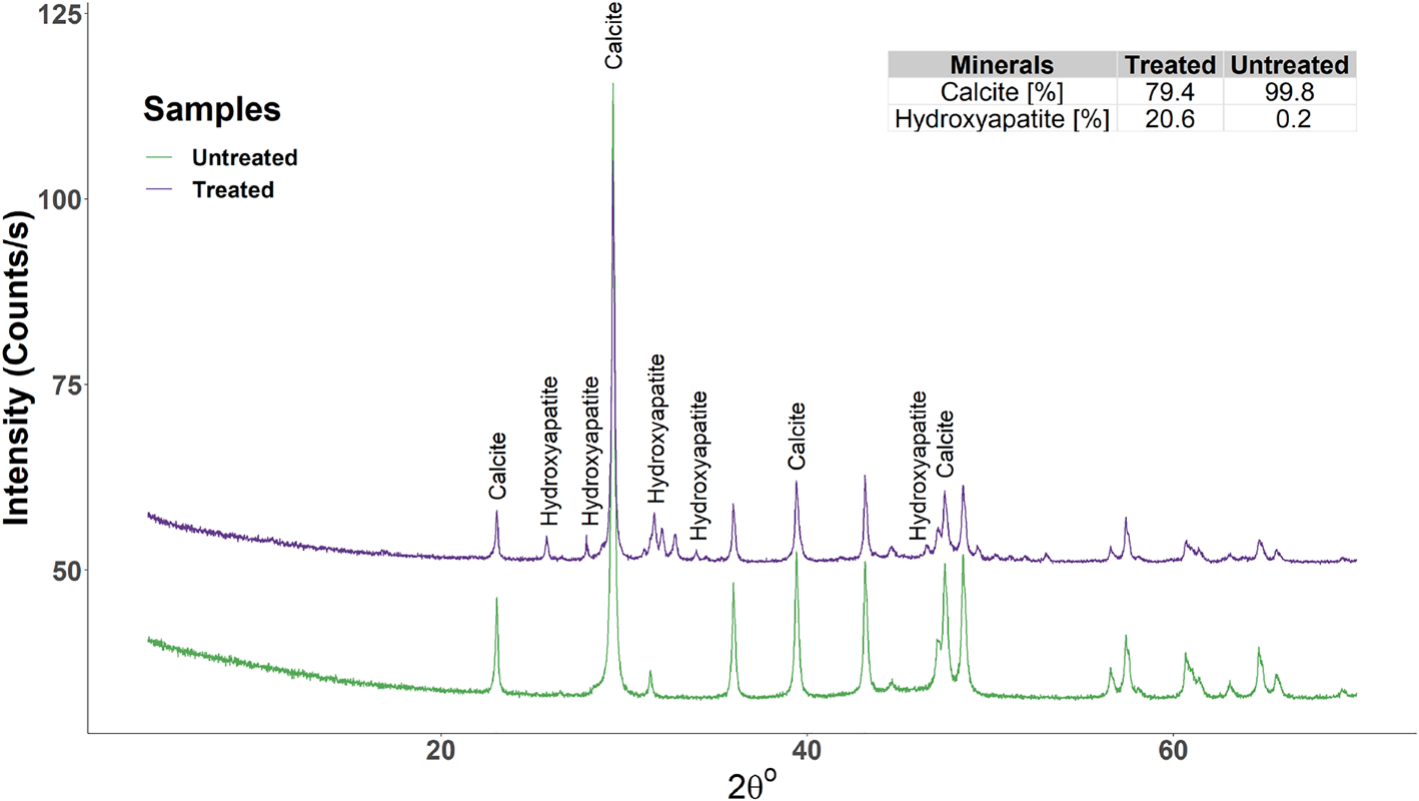
XRD spectra of treated and untreated specimens.

The mineral composition of rock specimens underwent significant changes after treatment, as was indicated by the XRD analysis comparing pre- and post-treatment spectra. The analysis showed that newly formed peaks were present in the spectra of chalk specimens after treatment with the DAP solution. The major peaks were located between angle positions of 30° and 35°, as well as around the 26–27° position (Fig. 4). According to previous studies, these positions correspond to hydroxyapatite minerals^62,63^.

**Figure 5:**
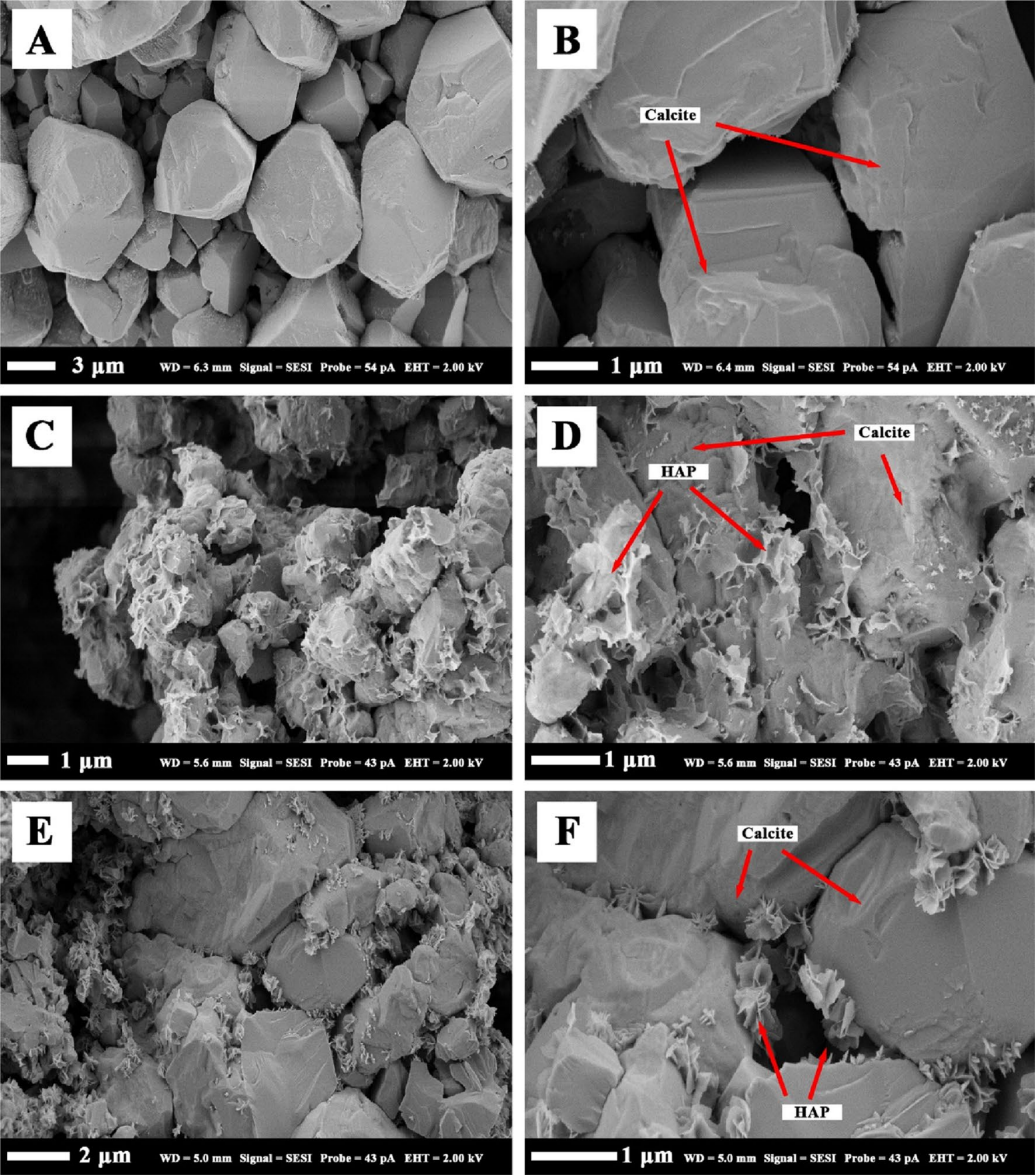
Morphology of the hydroxyapatite minerals formed at different temperatures: (**A**) untreated (× 3590), (**B**) untreated (× 14,760), (**C**) ambient (× 8700), (**D**) ambient (× 15,410), (**E**) 50 °C (× 6930), (**F**) 50 °C (× 18,800).

New minerals were clearly observed in the resultant SEM images. The minerals formed in a such way that they bonded multiple grains together, and their size, crystallinity, and nucleation pattern depended on the treatment temperature. At ambient temperature, the resulting structures were akin to intricate cobweb networks (Fig. 5C,D). At 50 °C, the structures were more compact and had a distinct roselike shape (Fig. 5E,F). The SEM images of the untreated chalk at 40 and 10 microns are shown in Fig. 5A,B, respectively.

### Compressive strength measurements

**Figure 6:**
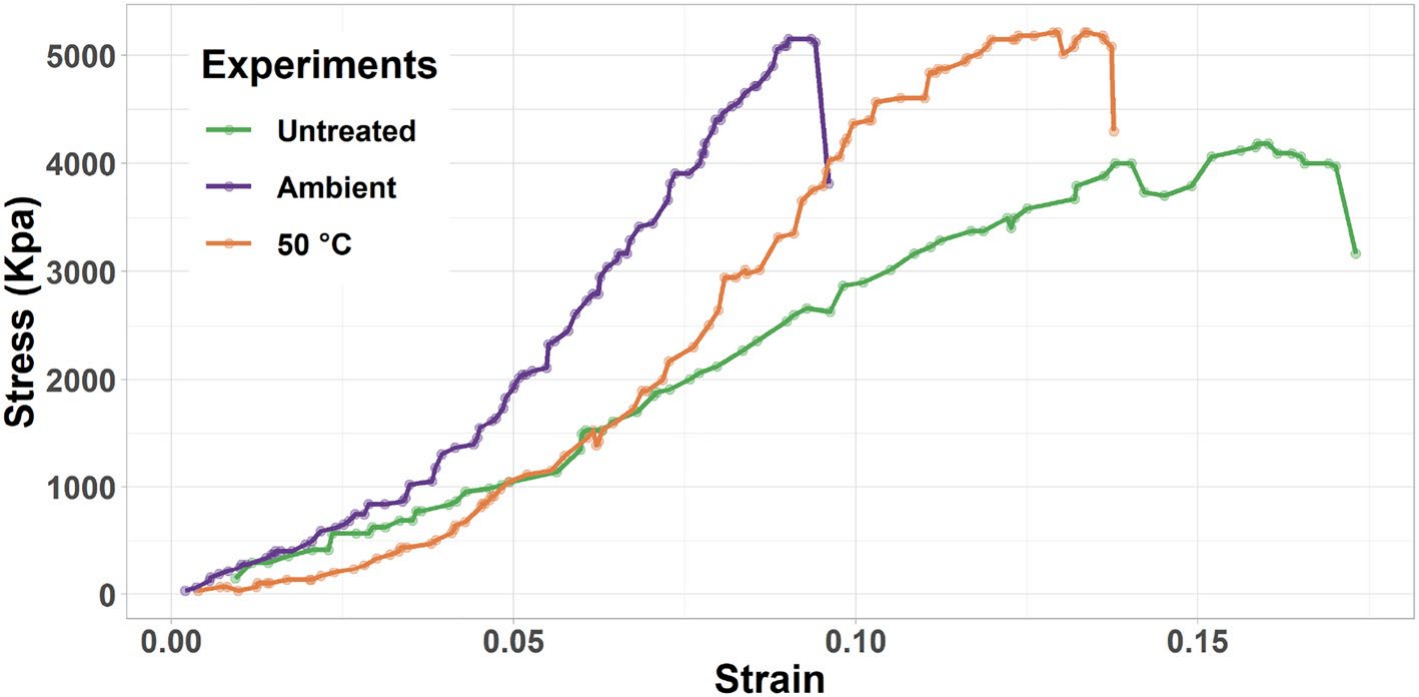
Reconstructed 3D views of samples.

Compressive force was applied to the samples until their failure. The maximum force that was applied before the failure of untreated, treated at ambient conditions, and treated at 50 °C temperature samples was 1547 N, 1834.3 N, and 1702 N, respectively. The CT—scan of the samples was taken at different times while the force was constantly applied to the samples. Figure 6 demonstrates the 3D rendered volume of the samples from micro-CT scans at initial, interim, and failure states. It can be noticed that the spreading of the crushed rock fragments is dependent on the maximum applied force (more spreading for treated samples).

**Figure 7:**
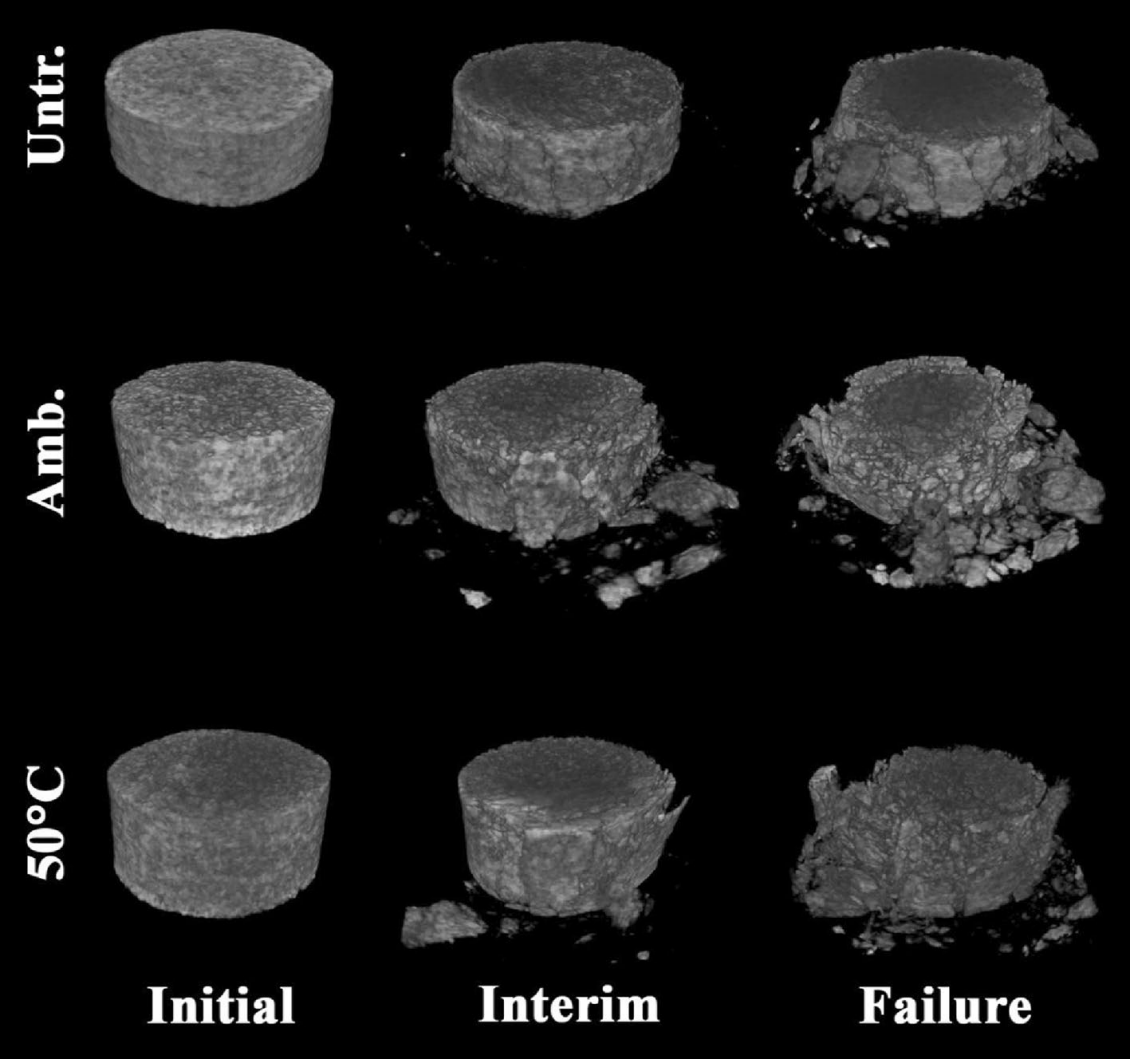
Stress–strain relationship for the tested specimens.

The applied force and deformation data obtained during experiments were used in the generation of stress–strain curves which are shown in Fig. 7. It can be noticed that samples behave in almost similar ways at lower stresses, however, samples treated with diammonium hydrogen phosphate possess less aggressive deformation at higher stresses compared to the untreated sample. Furthermore, the strength of the treated samples was found to be significantly higher than the strength of untreated samples. The strengths of untreated, treated at ambient conditions and 50 °C temperature samples were found to be 4.2 MPa, 5.13 MPa, and 5.21 MPa, respectively. As such, treated samples have around 22–24% higher compressive strength value compared to the untreated sample. Moreover, Young’s Modulus values were found from the stress–strain relationships for the samples and are presented in Table 2. It is also important to note that the treated samples possessed less ductility than the untreated samples. As ductility is related to the plastic deformation, i.e. sample’s hardness was improved.

**Table 2:** Calculated Young's Modulus values.

Sample	YM (MPa)	Comparison with untreated (%)
Untreated	26.5	–
Amb	57.1	116
50 °C	39.1	48

## Discussion

This work expanded the scope of the research on diammonium hydrogen phosphate (DAP) rock consolidation to include a different method of rock mechanical properties assessment, such as compressive strength measurements. Compression tests allowed for a more realistic assessment of the mechanical properties of the rock specimens at stresses similar to those existing downhole. The results of the experiments conducted in this research have demonstrated that the treatment of the samples with DAP solution creates improvements in the rocks’ mechanical properties which corroborates the findings of other authors^15,37,47,48,64^. While most of the previous research works were focused on assessing the changes in the hardness of the carbonate specimens, the compression tests conducted in this research have shown that the overall strength of the samples improves due to treatment with the DAP solution, and this improvement is tentatively ascribed to temperature treatments. Furthermore, the results once again confirmed that DAP is one of the most effective consolidating agents for hardening and strengthening carbonate rocks, as observed by other authors^21,36,43,45,65^.

The highest improvement was documented for samples treated at 50 °C. However, the difference in strength values achieved at ambient temperature (5.13 MPa) and 50 °C (5.21 MPa) was not significant. This can be explained by observations from a previous study that focused on analyzing changes in hardness in carbonate samples after the DAP treatment^14^. The study found that 50 °C does not result in a substantial improvement compared to ambient conditions, while much higher temperatures, such as 80 °C, lead to more significant increases in hardness. It is possible that there is a threshold temperature at which the reaction of DAP with calcium carbonate proceeds more intensively, resulting in a more significant improvement in the rock's mechanical properties. In addition to this, the current study has also confirmed observations documented in previous research^37,66^ that the specimens’ Young's moduli or stiffnesses are also improved due to the DAP treatment. Young’s modulus improvement was more significant in the case of ambient temperature treatment (around 115%). A more significant improvement in the sample's Young’s modulus during ambient temperature treatment can be attributed to the heterogeneous nature of the carbonate samples. One constraint of the current research is that strength measurement is a destructive technique, and therefore, strength cannot be assessed for the same sample before and after treatment. Even though the samples used in this research were of the same lithology and were cut from sister rock samples, it is still possible that their initial mechanical properties did not closely match those of the untreated reference sample. However, a previous study that focused on Young's modulus measurements has demonstrated that higher temperatures result in more significant changes in Young's modulus^14^.

In addition to the improvement in Young's modulus and strength, the treated samples exhibited a reduction in ductility. This reduction in ductility indicates that the samples undergo less plastic deformation under applied stresses. The plasticity of rock samples is one of the reasons for wellbore instability issues, such as wellbore closure or narrowing^67^. Since some chalk formations exhibit severe plastic behavior, treating them with DAP can reduce instability associated with such behavior. However, in the context of wellbore collapse or fracturing, the most critical parameter of the rock is its strength. Higher strength implies that the rock can withstand higher stresses before failure occurs, and it has been demonstrated that DAP treatment can improve the rock's strength.

Furthermore, the present study investigated the mineral composition of rock specimens before and after treatment, as well as the subsequent changes observed through XRD and SEM analyses. The XRD analysis revealed significant alterations in the mineral peaks of the chalk specimens following treatment with the DAP solution. Notably, the emergence of newly formed peaks in the spectra, predominantly between angle positions of 30° and 35°, and around the 26–27° position, suggested the presence of hydroxyapatite minerals, which aligns with previous research findings^62,63^. In addition to XRD, SEM images clearly demonstrated the formation of hydroxyapatite minerals, which acted as bonding agents between multiple grains. It was reported in the literature that the formation of the hydroxyapatite minerals inside the rock samples does not significantly affect its porosity^34,43^ but may moderately reduce its permeability^48^.

The morphology and structure of formed hydroxyapatite minerals varied depending on the treatment temperature. At ambient temperature, intricate cobweb networks were observed, while at 50 °C, more compact and roselike structures were prevalent. The SEM micrographs provided evidence of the newly formed minerals on the surface and within the samples, emphasizing their coating-like nature. The nanoscale observations further revealed changes in size, shape, crystallinity, and nucleation patterns of the new hydroxyapatite minerals due to temperature variations, consistent with previous studies^68,69^. Ambient temperatures yielded complex cobweb networks, while higher temperatures resulted in compacted structures resembling roselike formations.

It is also important to discuss some potential limitations of the conducted study. As was mentioned previously, strength measurement, being a destructive technique, prevents the assessment of the same sample's strength before and after treatment. Despite using samples of the same lithology cut from sister rock samples, there is still the possibility that their initial mechanical properties differed from those of the untreated reference sample. To obtain a clearer trend in the changes in the mechanical properties of DAP-treated samples under different conditions, future studies should include more samples to statistically account for the initial mechanical property disparities resulting from the heterogeneous nature of carbonate rocks. Furthermore, the strength testing of the samples was conducted under ambient conditions, which may not accurately represent the conditions occurring downhole. Therefore, future studies should involve testing the strength of samples treated at higher temperatures, as well as varying pressure and solution concentrations, to enhance the current understanding and simulate reservoir conditions. Moreover, it has been shown that higher temperature and pressure conditions in addition to the initial concentrations of the treatment solution can result in a more pronounced improvement in the mechanical properties of carbonate specimens^43,46,48^. Also, subsequent examination of carbonate rocks can be expanded to include larger specimens and encompass aspects like tensile properties, addressing all potential deformation scenarios that contribute to wellbore instability problems. Overall, the positive findings from this research suggest diammonium hydrogen phosphate (DAP) consolidating agent can be a good candidate for strengthening the chalk formations and addressing the wellbore instability issues.

## Conclusions

This study involved the strength testing of the chalk specimens treated with diammonium hydrogen phosphate (DAP) at ambient (25 °C) and 50 °C temperature conditions. Moreover, the samples’ mineralogy was investigated after the DAP solution treatment to observe the presence of hydroxyapatite minerals. XRD and SEM techniques have shown that treated with DAP samples contained hydroxyapatite minerals and their crystallinity and morphology depended on the treatment temperature. The treated samples’ compressive strength was contrasted with the strength of untreated specimens to study whether the DAP solution has any impact. The strength measurements have demonstrated that both treatments resulted in the improvement of specimens’ strength by around 22–24%. In addition, the treated samples demonstrated up to 115% higher Young’s Modulus values in comparison to untreated ones. Improving the strength and Young’s Modulus of the reservoir carbonate rocks through DAP solution treatment can help in the solving the wellbore instability issues frequently arising in carbonate formations.

## Data Availability

The datasets used and/or analysed during the current study are available from the corresponding author on reasonable request.
